# Congenital Pulmonary Airway Malformation (CPAM): A Case Report, Jimma University Medical Center, Southwest Ethiopia

**DOI:** 10.4314/ejhs.v31i4.27

**Published:** 2021-07

**Authors:** Habtamu Sime, Gersam Abera, Abera Mengistu, Sabona Lamessa

**Affiliations:** 1 Department of Pediatrics and Child Health, Jimma University, Ethiopia; 2 Department of Surgery, Jimma University, Ethiopia

**Keywords:** Case report, Pulmonary Airway Malformation, Multiple Cysts, pulmonary adenoid malformation

## Abstract

**Background:**

Congenital pulmonary airway malformation is a very rare congenital cystic lung disease that presents in 0.004% of all pregnancies and constitutes <25% of all congenital pulmonary anomalies in children. Respiratory distress is a major concern in these patients.

**Case Details:**

Here in, we report an 8 month old girl presenting with exacerbation of fast breathing of three days duration. Chest X-ray showed hyper lucent right lung with significant shift of mediastinum to the left side, flattening of the diaphragm on the right side and compression of the left lung. Computed tomography scan of the chest revealed multiseptated cystic mass on the right lung measuring 8.9cm by 6.9cm. After receiving treatment for pneumonia, surgical excision of the mass was performed and biopsy showed congenital pulmonary airway malformation type1. The infant died on 40^th^ postoperative day from uncontrolled hospital acquired infection.

**Conclusion:**

When a child has respiratory distress, congenital pulmonary airway malformation could be considered after common pathologies are ruled out. Surgical excision, which is the treatment of choice, is recommended to make a definite diagnosis and exclude hidden malignancies.

## Introduction

Congenital pulmonary airway malformation (CPAM) is focal developmental malformation of the lung, usually forming a single, large, multiloculated, cystic structure. It presents in 0.004% of all pregnancies and constitutes <25% of all congenital pulmonary anomalies (1–3). Its estimated incidence is 1 in 25,000 to 1 in 35,000 live births. It is not associated with race, age, maternal exposure to any given factor or genetic factor (1). Its usual postnatal presentation is respiratory distress in the newborn period (4). It can spontaneously regress, increase in size or cause nonimmune hydrops fetalis. The mortality rate from CPAM is12.5% (5). The postnatal management of CPAM depends on whether the patient has respiratory distress or is asymptomatic (5).

## Case Summary

**Figure 1:**
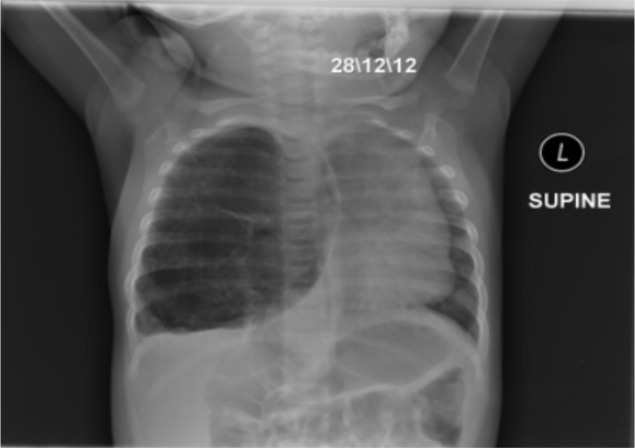
Chest X-ray of an 8-month-old infant with congenital pulmonary malformations N.B: The date on the CXR is according to Ethiopian calendar.

**Figure 2 (A and B):**
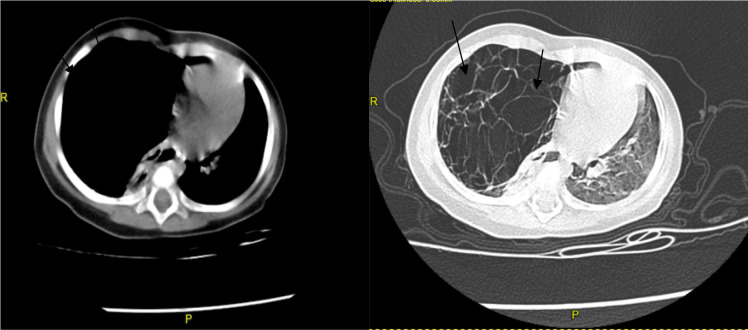
Computed tomography scan an 8-month-old infant with congenital pulmonary malformations.

This is an 8-month-old infant girl admitted to pediatric emergency ward for fast breathing since age of five months. For this problem she was treated repeatedly at private clinic and health center, but did not get any improvement and was referred to our center for acute exacerbation of her condition. Despite aforementioned problems, her developmental milestones are not affected. Prenatal ultrasonography and postnatal physical examination were normal according to parental report. She was born in hospital after an uneventful 38 weeks of gestation. On admission, she was dyspneic with temperature 37.6°C, respiratory rate 60/minute, heart rate 124/minute, SPO2 89% with room air and all her anthropometric measurements were normal. She had dullness on the right lower 2/3 of the posterior right lung and decreased breath sounds on the right anterior and posterior lung fields. She was treated for severe pneumonia but there was no improvement after 48 hours of treatment. Hence, chest X-ray (CXR) was taken and showed hyper lucent right lung with significant shift of mediastinum to the left side, flattening of diaphragm on the right and compression of the left lung (Figure 1). Computed tomography (CT) scan showed multiseptated cystic mass on the right aspect of lung and displacement of the mediastinum and heart to the left side with an index of multiseptated right side cystic lung mass likely CPAM (Figures 2A and B). After receiving treatment for pneumonia until her fever subsides, the infant underwent right lung lobectomy. The resected lung tissue was subjected for biopsy which showed congenital cystic adenomatoid malformation type 1 (Figure 3). The infant was admitted to pediatric intensive care unit (PICU)on immediate post-operative day and put on mechanical ventilation and other supportive care. Her SPO2 was 95%with 45% of oxygen. On fifth post-operative day, the infant had fever and started to desaturate. For this, blood culture and CXR were done. Blood culture showed klebsiella oxytoca, which was sensitive only for meropenem and CXR showed bilateral opacities. The infant was treated with meropenem for the infection and with other respiratory support during her stay. However, despite this, the infant died on her 40^th^ post-operative day. Autopsy was not done to confirm the cause of death.

**Figure 4:**
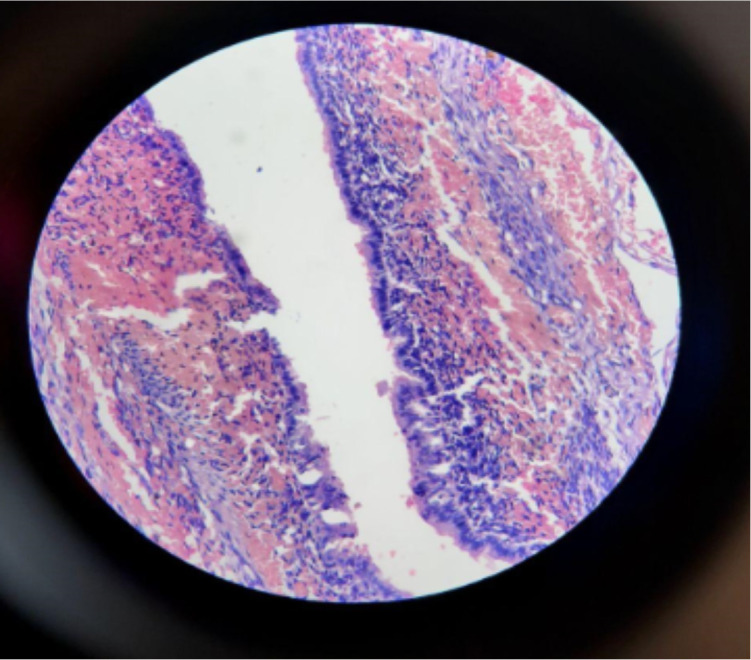
Microscopic pictures of biopsy from lobectomy specimen, showing multiple cystic spaces lined by cuboidal to ciliated pseudo stratified columnar epithelia

## Discussion

Congenital cystic lung disease in children is rare with various clinical presentations. CPAMs result from abnormalities of branching morphogenesis of the lung. The different types of CPAMs originate at different levels of the tracheobronchial tree and at different stages of lung development, possibly influenced by in utero airway obstruction and/or atresia. The mechanisms resulting in CPAM formation are unknown but may include an imbalance between cell proliferation and apoptosis during organogenesis (1).

Pathologically, CPAMs are hamartomatous lesions that are comprised of cystic and adenomatous elements arising from tracheal, bronchial, bronchiolar, or alveolar tissue. Large lesions can compromise alveolar growth and development by compressing adjacent normal tissue (4).

CPAMs are equally distributed between the right and left lungs and can arise in all lobes. Lesions are usually limited to one lobe, but infrequently, they can involve multiple lobes (1).

CPAMs were previously known as congenital cystic adenomatoid malformations (CCAMs) which were divided into three major types based upon the size of the cysts and their cellular characteristics (predominantly bronchial, bronchiolar, or bronchiolar/alveolar duct cells). More than 65 % of CPAMs were type 1, while type 2 comprised 20 to 25 % and type 3 comprised 8 %(1).

Currently it is called CPAM and two additional types (0 and 4) were added. Type 0 arises from the trachea and type 4 lesions have alveolar/distal acinar origins. Each type of CPAM has distinct pathologic characteristics(2). Type 1 comprises 60 to 70% and originates from the distal bronchi or proximal bronchioles. Because there is well-differentiated tissue within the lesions, this type probably originates relatively late during embryogenesis. It is comprised of distinct thin-walled cysts 2 to 10 cm in diameter. The cysts are usually single but may be multiloculated. They are lined with ciliated pseudostratified columnar epithelium, which was found in our case. In 95% of the cases, only one lobe of the lung is involved and it has malignant potential. Its clinical presentation depends on the size of the cysts; if they compress the adjacent normal lung, cause respiratory distress in the neonate, mediastinal shift to the contralateral side, and flattening of the ipsilateral diaphragm, which was also true in our case(2). Type II lesions are associated with other congenital malformations. Type III, arising from acinar type of tissue, accounts for 5–10 % of the cases. Type IV may be associated with malignancy (2).

Patients with CPAM have different clinical presentations. Respiratory distress, caused by a ball-valve mechanism, is the most common mode of presentation seen in about 80% of cases(2). The management of CPAM depends on whether the patient has respiratory distress or is asymptomatic. In symptomatic patients, CPAM is treated by surgical resection, whereas for asymptomatic patients, close observation is important because some infants are asymptomatic immediately after birth but then become symptomatic as the cystic lung increase in size (2).

In conclusion, CPAM is a rare pathology. CPAM should be considered in infants who presented with respiratory distress and not responding to treatment after *common clinical conditions are ruled out.*
